# Effect of Graphene Oxide on the Properties of Porous Silicon

**DOI:** 10.1186/s11671-016-1264-5

**Published:** 2016-02-01

**Authors:** Igor B. Olenych, Olena I. Aksimentyeva, Liubomyr S. Monastyrskii, Yulia Yu. Horbenko, Maryan V. Partyka, Andriy P. Luchechko, Lidia I. Yarytska

**Affiliations:** Radioelectronics and Computer Systems Department, Ivan Franko National University of Lviv, 50 Dragomanov Street, 79005 Lviv, Ukraine; Physical and Colloidal Chemistry Department, Ivan Franko National University of Lviv, 6 Kyrylo and Mefodiy Street, 79005 Lviv, Ukraine; Solid State Physics Department, Ivan Franko National University of Lviv, 50 Dragomanov Street, 79005 Lviv, Ukraine; Electronics Department, Ivan Franko National University of Lviv, 107 Tarnavskyi Street, 79017 Lviv, Ukraine; Thermodynamics and Physics Department, Lviv State University of Live Safety, 35 Kleparivska Street, 79000 Lviv, Ukraine

**Keywords:** Porous silicon, Graphene oxide, Hybrid structure, Photoluminescence, Current–voltage characteristics, Impedance spectroscopy, 73.63.-b, 78.67.-n, 81.05.Rm

## Abstract

We studied an effect of the graphene oxide (GO) layer on the optical and electrical properties of porous silicon (PS) in hybrid PS–GO structure created by electrochemical etching of silicon wafer and deposition of GO from water dispersion on PS. With the help of scanning electron microscopy (SEM), atomic-force microscopy (AFM), and Fourier transform infrared (FTIR) spectroscopy, it was established that GO formed a thin film on the PS surface and is partly embedded in the pores of PS. A comparative analysis of the FTIR spectra for the PS and PS–GO structures confirms the passivation of the PS surface by the GO film. This film has a sufficient transparency for excitation and emission of photoluminescence (PL). Moreover, GO modifies PL spectrum of PS, shifting the PL maximum by 25 nm towards lower energies. GO deposition on the surface of the porous silicon leads to the change in the electrical parameters of PS in AC and DC modes. By means of current–voltage characteristics (CVC) and impedance spectroscopy, it is shown that the impact of GO on electrical characteristics of PS manifests in reduced capacitance and lower internal resistance of hybrid structures.

## Background

Graphene, a two-dimensional (2D) sheet composed of sp^2^-bonded single-layer carbon atoms with the honeycomb lattice structure, and graphene oxide (GO), which derives its name from the oxidation process of graphite, are known to be extensively researched materials. Graphene and graphene-based materials have been attracting great research interest with regard to their unique structural features—high surface area, flexibility, superior electric and thermal conductivity, and chemical stability [1–4]. Outstanding electronic and optical properties of graphene expand its applications in diverse field such as sensors, photodetectors, energy conversion, and storage devices. One of the promising methods for producing graphene is via chemical exfoliation and oxidations of graphite to produce GO followed by subsequent reduction. GO is therefore regarded as the most important precursor material.

The chemical composition of GO is classified into rich oxidized region where hydrophilic functional groups (i.e., epoxy and hydroxyl at the planar surface and carboxyl groups at the edges) are anchored to sp^3^ carbon atoms as well as pools of un-oxidized graphitic domains which consist of hexagonal aromatic chains of sp^2^-bonded carbon atoms [5, 6]. The chemical structure of GO renders amphiphilic character to the material [7, 8]. GO has received great interest because of its superior dispersion ability in water and electronic bandgap different with respect to graphene. Graphene and GO are appealing materials for different applications because they offer a wide palette of advantages compared to other materials [9–12].

Porous silicon (PS) is a perfect candidate to infiltrate GO into the substrate with a large specific surface area. PS is prepared by etching a single crystal with the formation of small cavities, resulting in a thick wall between the pores that may have a size of several nanometers [13, 14]. PS has a number of useful properties. An intense visible photoluminescence of PS, its anti-reflective properties and band gap increased due to the quantum confined effect in silicon nanocrystals give a perspective of PS use in optoelectronics and photonics [15, 16]. High chemical and adsorption sensitivity of large internal surface PS is actively exploited in the touch-sensing electronics and biomedical technologies [17–20].

Creating hybrid structures or composite materials allows to maximize the size effects and large specific surface areas of silicon and carbon nanoparticles [21–23]. Besides, an interaction between nanoparticles in the hybrid structures can lead to the appearance of new unique properties compared to those of the individual components. In particular, the modulation of fluorescent radiation was detected in PS–GO structures [24]. This effect is attributed to the interference phenomena occurring inside the PS layer. The PS-based photodetectors with surface coated by reduced GO have high sensitivity and quantum efficiency over a wide spectral range—from near UV to IR [25]. Owing to hydrophilic properties and high sensitivity to adsorbed gases [26], GO layers on the PS substrate are promising structures for humidity-sensing applications.

Hybrid structures based on PS and reduced GO are good candidates for electrode materials that are used for supercapacitor and lithium-ion battery applications [27, 28]. The porous structure of such nanomaterials can increase the electrical conductivity and reduce the transfer resistance of Li ions. Hybrid nanostructures exhibit high specific capacity and good cycling stability.

In the present research, GO was used for the modification of PS layers in order to improve its processability. Due to the high stability of GO, special attention was focused on exploring its role in the passivation of PS surface. We investigated the effect of GO on the electrical and photoluminescent properties of PS.

## Methods

PS was manufactured by means of photoelectrochemical etching performed in ethanol solution of hydrofluoric acid (the volume ratio of the components HF:C_2_H_5_OH = 1:1) on single-crystalline silicon substrates with the typical thicknesses of 400 μm and the crystallographic orientation (100). The silicon substrates were the n-type conductivity (n-Si), with the specific resistance of 4.5 Ω · cm. In order to obtain homogeneous layers, gold films were preliminarily deposited on a back surface of the substrates with the aid of a thermo-vacuum technique. These films served also as contacts for further measurements. We used an experimental setup in which a silicon sample (anode) and a platinum cathode were placed into a fluoroplastic cell. The anodic current (the density of 30 mA/cm^2^) was constant throughout the etching time (about 10 min). To ensure availability of holes in the surface layer of n-Si, which were necessary for occurrence of anodic reactions and formation of the PS, the working surface of a silicon plate was irradiated with white light during the whole process of electrochemical etching. Formation of narrow pores, directed towards the inside of the crystal, takes place during the anodic etching of silicon in ethanol solution of hydrofluoric acid. Pores gradually expand until the walls are partially etched. As a result, residuals of the walls, or the so-called quantum wires, remain. Under the technological conditions mentioned above, macroporous silicon layers were formed. After electrochemical processing, the working surface of the sample was washed with distilled water and dried in air.

To obtain the PS–GO nanocomposite, graphene oxide, produced by Biotool (Germany) in the form of an aqueous suspension (concentration of basic substance was 2 mg/ml), was used. The GO suspension was dispersed using ultrasonic processing and deposited onto a PS surface and then dried at the room temperature during 48 h under dynamic vacuum.

PS–GO hybrid structure was characterized by atomic-force microscopy (AFM) “Solver Pro” and scanning electron microscopy (SEM) REMMA-102-02 (Selmi, Ukraine). Molecular structure of the experimental samples was explored using the Fourier transform infrared (FTIR) spectroscopy. The transmittance spectra were measured with an “Avatar” spectrometer in the wave number region of 400–4000 cm^−1^. To identify the absorption bands of the PS and PS–GO composites, the literature data [7, 29–32] were considered.

The electrical properties of the obtained structures were investigated in both DC and AC regimes. The electrical parameters of the samples were measured experimentally with standard techniques. The electrical current flowed through the structures in the direction perpendicular to their surfaces. The current–voltage characteristics (CVC) were measured using AFM tip which was positioned on the PS surface or GO surface plate. Impedance spectroscopy of the experimental samples was performed using R, L, C measuring device E7-20 (Belarus) at the room temperature in the frequency range of 25 Hz–1 MHz. In this case, silver clamping contacts with the diameter of 3 mm were used.

Optical-luminescent properties were studied using СМ2203 spectrofluorometer (Solar, Belarus). Optical absorbance spectra of the GO film on the glass substrate were measured in the 220–1000 nm range. Photoluminescence spectra were registered in the 450–800 nm range using UV light excitation (*λ* = 330 nm) at ambient temperature.

## Results and Discussion

### Characterization of the PS–GO Hybrid Structure

**Fig. 1:**
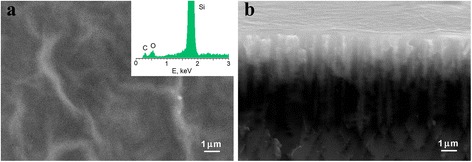
SEM images of the surface (**a**) and cross sections (**b**) of the PS–GO structures. *Inset*: X-ray surface microanalysis of the PS–GO structures

Analysis of the surface and the cross section of the PS–GO structures was carried out using SEM methods in modes of elastically reflected electrons and X-ray microanalysis. As one can see from Fig. 1, the formation of narrow pores was observed. The pores were oriented perpendicular to the silicon surface. Average pore diameter is within the range of 100–1000 nm (see Fig. 1b). GO formed a film on the surface of PS and partially penetrated into the pores. The X-ray surface microanalysis (EDS) of the hybrid structures found the traces of silicon, carbon, and oxygen. This fact proves that the surface of PS was covered by GO.

**Fig. 2:**
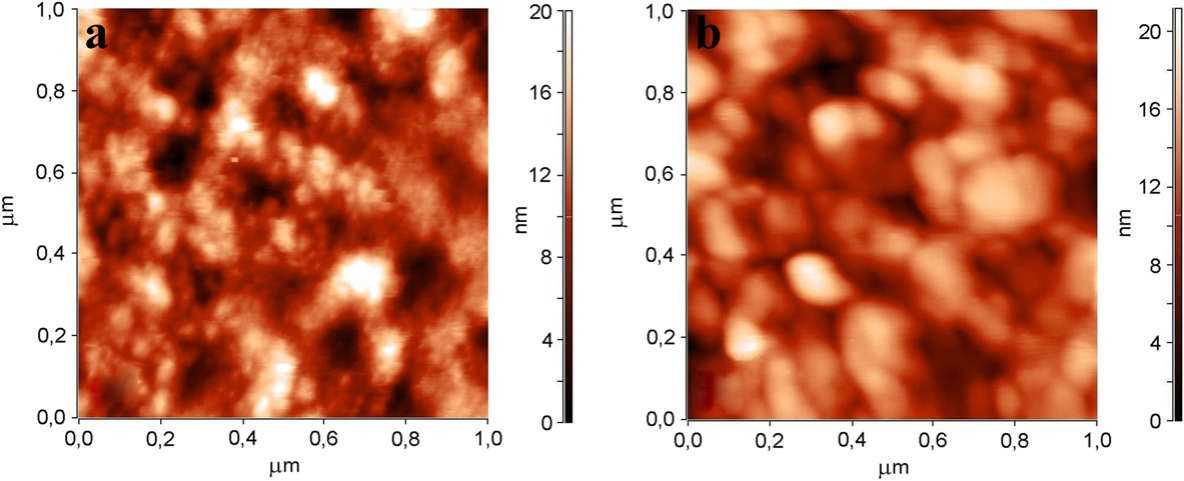
Two-dimensional AFM micrograph of the PS (**a**) and PS–GO (**b**) structures

The surface topography of the PS and PS–GO structures was studied using AFM in the tapping mode. Figure 2 shows the two-dimensional micrograph of 1 × 1 μm^2^ area of the experimental samples. According to the SEM and AFM data, the PS surface had the form of vertical nanowires (cylindrical nanocrystals), with the dimensions from several nanometers to tens of nanometers in the cross section. Walls between pores were well passivated by the GO film formed on the surface of PS.

**Fig. 3:**
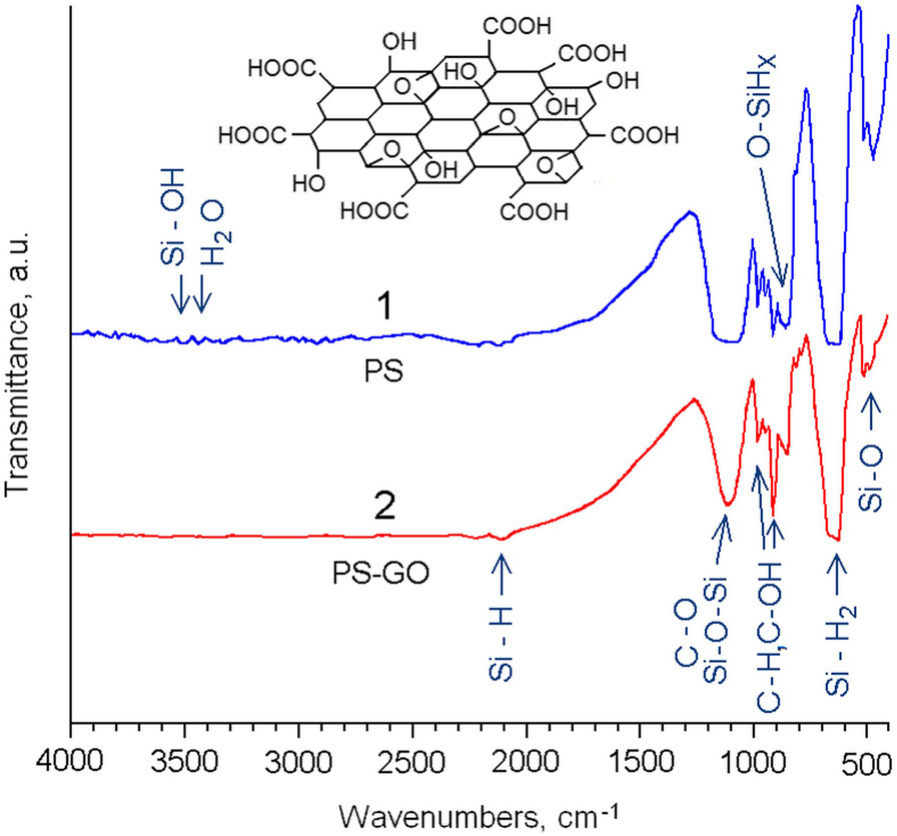
FTIR spectra of the PS (*1*) and PS–GO (*2*) structures. *Inset* shows the chemical structure of GO

To identify the components of the hybrid PS–GO structure, we measured their FTIR spectra. The analysis of the IR spectra of PS has shown that most of the absorption bands are related to molecular complexes containing hydrogen and oxygen (Fig. 3).

Most prominent are IR bands in the 620–660 cm^−1^ range corresponding to bending Si–H_2_ mode and the absorption band located at 1100 cm^−1^ that can be related to valence Si–O–Si vibrations [29, 30]. The band at 460 cm^−1^ is usually ascribed to deformation vibrations of the Si–O group [31]. Besides, the IR spectra of the PS include the absorption bands within the 860–1000 cm^−1^ range and 2100–2150 cm^−1^ range. These bands are characteristic for hydrogen-containing molecular complexes: O–SiH_*x*_ (*х* = 1,2) and Si–H, respectively. In the range of 3400–4000 cm^−1^ in the FTIR spectra of PS, a number of absorption bands of low intensity were observed. Usually, the absorption in this spectral region is associated with Si–OH groups and water molecules adsorbed by PS [30, 31].

A comparative analysis of the FTIR spectra for the PS and PS–GO composites reveals a transformation of the absorption band within 1100 cm^−1^ that can be due to С–О groups (see [7]) resulting from the passivation of the PS surface by GO films. Besides peak located at 860 cm^−1^ that originates from deformational vibrations Si–OH, we observed the bands in the region from 930 to 1000 cm^−1^, characteristic for С–H and C–OH complexes [22, 32] (see Fig. 3). It should be noted that the weak absorption of carbon complexes in the IR spectra of PS may be due to interaction of silicon with ethanol during the process of etching.

### Investigation of the Optical-Luminescent Properties of the PS–GO Structure

**Fig. 4:**
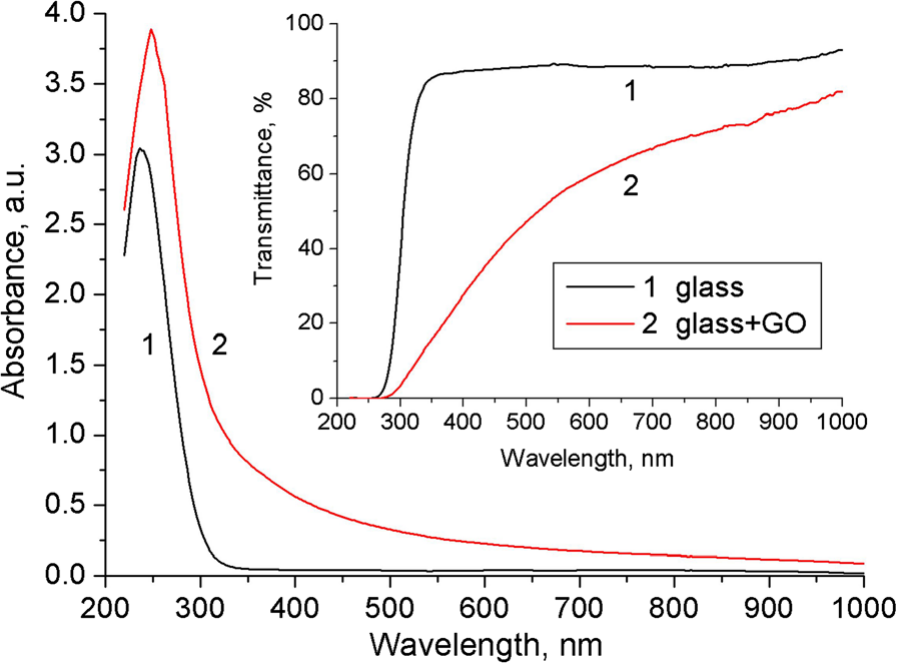
UV–vis absorbance and transmittance spectra of the glass substrate (*1*) and GO on the glass substrate (*2*)

The absorbance and transmittance spectra of GO on the glass substrate is shown in Fig. 4. The decreasing transmittance of the GO films compared to the glass substrate may be caused by the additional absorption and scattering of light by GO. The peak at 248 nm for GO is due to the *π* → π* transition of the C=C bonding, which is similar to the value reported in [7, 33]. Meanwhile, the light absorption in the near UV and blue part of the studied spectral range may be attributed to *n* → π* transition of the carbonyl groups [7]. The GO films have high optical transmittance in the visible region of the spectrum. This allows their use as a protective coating and window for the luminescence radiation generated in the PS. In addition, the deposited films of GO are transparent enough to excite the PL.

**Fig. 5:**
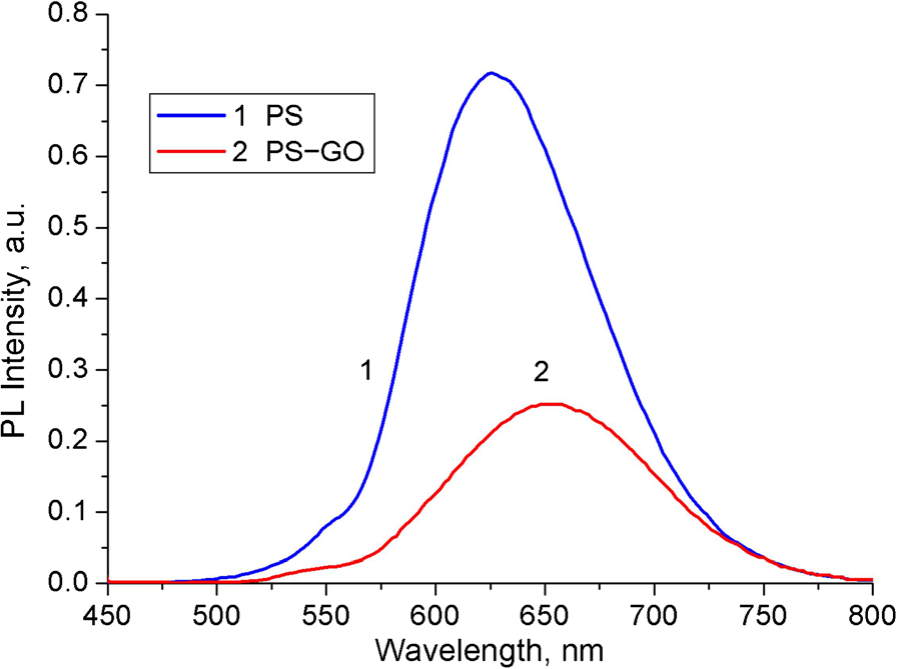
PL spectra of the PS (*1*) and PS–GO (*2*) structures

Figure 5 shows the PL spectra of the PS and PS–GO structures. The PL spectrum of PS shows a very intense emission peak at around 625 nm. A visible photoluminescence band of PS is a superposition of radiative processes resulting from the recombination of electron–hole pairs excited in silicon nanocrystals of different sizes. Electronic spectrum of such nanocrystals is modified due to the quantum confinement effect [15, 34]. Therefore, the photoluminescence band is broad and the peak position can be tailored by changing the technological route of PS preparation. This intense PL signifies the good quality of the PS matrix. GO has an effect on the visible PL peak position, shifting it from 625 to 650 nm. A possible reason for the spectral shift of the luminescence maximum is the interaction of the GO film with the centers of radiative recombination in the PS nanocrystals. Reducing the intensity of PL in the PS–GO structure may be due to the absorption of radiation by the GO layer.

### Investigation of the Electrical Properties of the PS–GO Structure

**Fig. 6:**
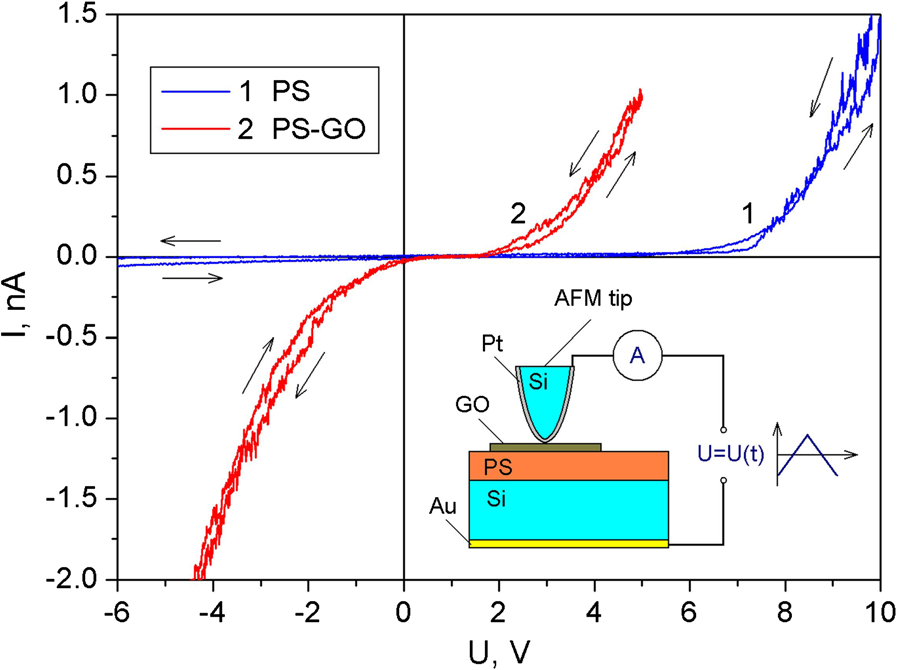
CVC of the PS (*1*) and PS–GO (*2*) structures. *Inset*: scheme of the CVC measurement for experimental structures, performed using the AFM tip

CVC of experimental structures based on PS, measured at room temperature using AFM probe, are shown in Fig. 6. The initial sample of PS was characterized with rectifier CVC, which can be caused by the Schottky barrier at the contact of the probe and PS nanostructures.

Deposition of the GO on the surface of the PS film changed the CVC to varistor-like, indicating the existence of several potential barriers in such structure. Nonlinear CVC can be caused by contact phenomena, electric barriers in the porous layer and on the borders of the PS–silicon substrate and PS–GO, or Poole–Frenkel effect [18, 35]. During the measurements, negligible hysteresis of CVC was found when changing the voltage from negative to positive values and vice versa. The reason for the observed hysteresis can be non-equilibrium filling of surface states in PS, which exchange electrons with the semiconductor [36].

**Fig. 7:**
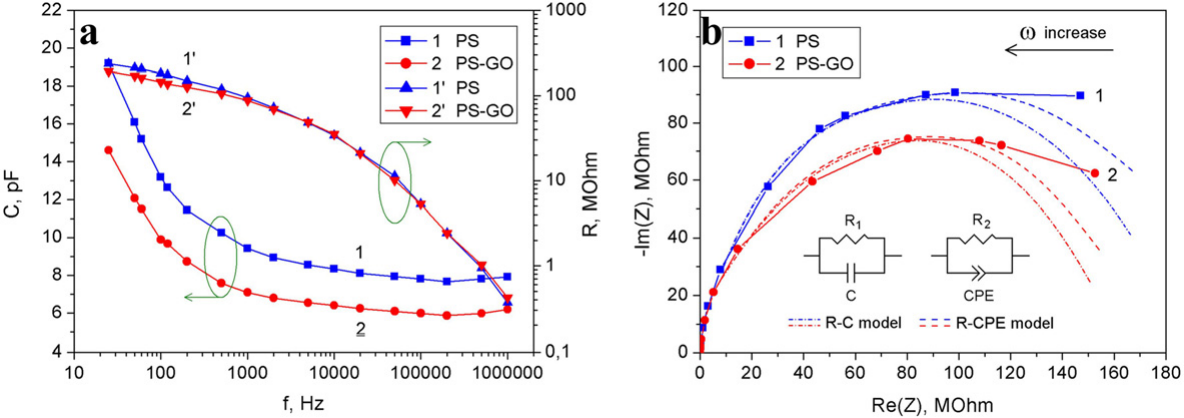
**a** Frequency dependence of the electrical capacitance (*curves 1* and *2*) and the resistance (*curves 1'* and *2'*) of the PS (*1*, *1'*) and PS–GO (*2*, *2'*) structures. **b** Nyquist plots of the PS (*1*) and PS–GO (*2*) structures. *Inset*: equivalent circuit diagram of PS-based structures

Impedance spectroscopy is a powerful technique that provides important information regarding the processes of charge transfer in the experimental structures [37, 38]. By means of impedance spectroscopy, it was found that experimental samples show a decrease in electrical capacitance and internal resistance with increase of the frequency (Fig. 7a). The PS–GO structures are characterized by lower capacitance as compared to PS in the range of 25 Hz–1 MHz and lower resistance in the low-frequency range.

The experimental dependence indicates the different dispersion of capacitance in different frequency ranges. Low-frequency dispersion is likely associated with the transfer of charge through the barriers of the PS nanocrystals. The complex frequency dependence of the capacitance can be related to the movement of massive electroactive particles such as ions of hydrogen as well as to other factors: hopping mechanism of charge transport via the localized states in PS or the recharge of capture levels.

Figure 7b presents Nyquist plots of the PS and PS–GO structures in coordinates of the complex plane *Z*_Re_–*Z*_Im_. To interpret the impedance of PS-based structures, two equivalent circuit models were constructed. Within the first model, the porous layer can be regarded as the composition of parallel-connected capacitor with the capacitance *C* and resistor with the resistance *R*_1_ [39]:

$$ Z\left(\omega \right)=\frac{R_1}{1+j\omega {R}_1C} $$

As it is evident from Fig. 7, the contribution of active resistance of the silicon substrate and the supply contacts is negligibly small (real axis value at *ω* = ∞ intercept is zero within the error of the experiment). Therefore, we do not consider this resistance in the construction of impedance models for experimental structures.

In the second model, the resistive-capacitive properties of the system are described using the constant phase element (CPE) in the equivalent circuit (see Fig. 7b). After replacing the capacitor by this element, the expression for the impedance of the structures based on PS will be the following [38]:

$$ Z\left(\omega \right)=\frac{R_2}{1+{\left(j\omega \right)}^n{R}_2Q}, $$

where *R*_2_ is the resistance, *Q* is the element of constant phase, and *n* characterizes the heterogeneity of the electrical properties of the structure (−1 ≤ *n* ≤ 1). In case *n* = 1, CPE element corresponds to the pure capacitance, and when *n* = 0, CPE element transforms into the simple active resistance. Parameters of the approximation of the impedance spectra of the PS and PS–GO structures are shown in Table 1.

**Table 1:** Parameters of the approximation of the impedance spectra of the PS and PS–GO structures

Structure	Parameters of approximation				
	*R*-*C* model		*R*-CPE model		
	*R*_1_, MΩ	*C*, pF	*R*_2_, MΩ	*Q* × 10^−11^	*n*
PS	176	8.95	197	1.69	0.944
PS–GO	155	6.68	166	1.20	0.949

CPE is a generalized and universal element widely used in the impedance modeling of fractal systems, which include PS-based structures [38, 40]. Considering the slight deviation of the obtained parameter *n* from 1, the interpretation of the impedance of experimental samples using capacitance element gives satisfactory results.

Deposition of the GO particles on the surface and incorporation in the pores of PS caused the drop in the internal resistance of the PS–GO structure from 197 to 166 MΩ (*R*-*C* model) or from 176 to 155 MΩ (*R*-CPE model).

## Conclusions

The hybrid PS–GO structures were created by the method of electrochemical etching of silicon wafer and deposition on the PS layer of GO prepared from water dispersion. It was found that GO formed a film on the surface of PS and partially penetrated into the pores. The effect of the GO layer on the luminescent and electrical properties of PS was studied using comprehensive studies. It was found that the GO film passivates the surface of PS and also is sufficiently transparent to allow excitation and emission of PL. In addition, GO modified the PL spectrum, shifting the emission maximum for Δ*λ* = 25 nm to lower energies. Deposition of the GO on the surface of the porous layer led to the changes of the electrical parameters of PS in AC and DC modes. Change of the character of CVC from rectifier-like to varistor-like can be caused by the appearance of new electric barriers in the hybrid nanosystems. The complex nature of the dispersion of electrical capacitance in PS-based structures was established using the method of impedance spectroscopy. Observed behavior of the dispersion is caused by the features of transport and relaxation of charges in disordered systems. Impact of the GO on electrical characteristics of PS manifests in reduction of the capacitance and internal resistance of the hybrid structures.

Proposed patterns and found features of the emissive and transport processes in the structures of PS–GO expand the knowledge about the properties of the interface between oxidized graphene and silicon nanocrystals that may be useful in the development of new photodetectors and gas sensors, in particular the sensors of humidity.
